# Coupling carbon nanomaterials with photochromic molecules for the generation of optically responsive materials

**DOI:** 10.1038/ncomms11118

**Published:** 2016-04-12

**Authors:** Xiaoyan Zhang, Lili Hou, Paolo Samorì

**Affiliations:** 1 ISIS & icFRC, Université de Strasbourg & CNRS, 8 allée Gaspard Monge, Strasbourg 67000, France

## Abstract

Multifunctional carbon-based nanomaterials offer routes towards the realization of smart and high-performing (opto)electronic (nano)devices, sensors and logic gates. Meanwhile photochromic molecules exhibit reversible transformation between two forms, induced by the absorption of electromagnetic radiation. By combining carbon-based nanomaterials with photochromic molecules, one can achieve reversible changes in geometrical structure, electronic properties and nanoscale mechanics triggering by light. This thus enables a reversible modulation of numerous physical and chemical properties of the carbon-based nanomaterials towards the fabrication of cognitive devices. This review examines the state of the art with respect to these responsive materials, and seeks to identify future directions for investigation.

## Abstract (web summary)

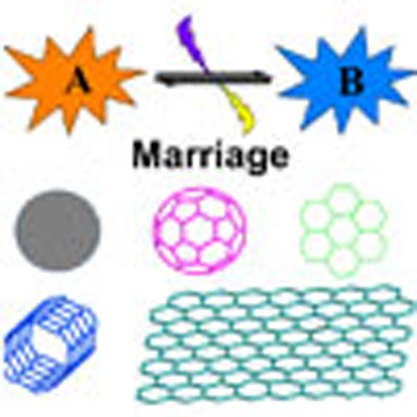
The marriage of photochromic molecules with the rapidly expanding portfolio of nanocarbons is providing new multifunctional and responsive nanomaterials. Here, the authors review recent progress in such materials' fabrication and their possible implementations, and suggest future directions of study.

Carbon is the fourth most abundant element in the universe by mass and it forms a vast number of compounds, more than any other chemical element. It assembles into several allotropes that hold extraordinary yet markedly different chemical and physical properties^1^,^2^,^3^,^4^,^5^,^6^,^7^, including their structure and geometry, optical, mechanical, electrical/electronic properties and stability, making them key building blocks for numerous applications in materials science and nanotechnology^8^,^9^,^10^,^11^,^12^,^13^,^14^,^15^,^16^,^17^,^18^. A common characteristic of fullerene, carbon nanodots, carbon nanotubes (CNTs) and graphene (including large polycyclic aromatic hydrocarbons) is the ability to functionalize them covalently and non-covalently with specific moieties, thereby improving their solubility in liquid media and imparting them with enhanced/additional properties. Among various moieties, the functionalization of carbon-based systems with building blocks that can respond to external stimuli enables the generation of smart/dynamic carbon-based nanomaterials, by offering additional remote controls (for example, light, mechanical pressure, pH, electric/magnetic fields and so on) to modulate their properties^19^,^20^,^21^. Among various stimuli, light is a perfect choice since it features high spatio-temporal resolution and it is non-invasive over a wide range of wavelengths. Furthermore, the possibility to tune their wavelength and intensity guarantees a wealth of solutions when the photochromic systems are intelligently designed.

Photochromic molecules can undergo reversible photo-triggered isomerization between (at least) two (meta)stable states. Hitherto, a variety of photochromic molecules have been designed and synthesized, using not only E/Z isomerization, but also valence isomerization, cycloadditions, tautomerizations and so on. Depending on the type of chemical processes involved, photochromic molecules can generally be divided into several classes^22^: (1) pericyclic reactions including electrocyclizations like spiropyrans/-oxazines, fulgides and diarylethenes, or cycloadditions in aromatic compounds; (2) E/Z isomerizations such as azobenzenes, stilbenes and so on; (3) intramolecular hydrogen/group transfer such as anils and polycyclic quinones; (4) photo-induced bond cleavages such as triarylmethanes and perchlorotoluene; (5) electron transfers (redox) such as viologens. Among all photochromic molecules, the most widely used are azobenzenes^23^, diarylethenes^24^, spiropyrans/-oxazines^25^ and stilbenes^26^. Among these four, diarylethenes can be switched between the two states photochemically or electrochemically, whereas the other three molecules can be reverted either photochemically or thermally (Fig. 1). The basic properties of the above mentioned photochromic molecules are summarized in Table 1. The switching behaviour, including rate of the isomerization and stability of the different isomers, depends on several factors such as their surrounding environment, substituents and so on.

The functionalization of carbon-based nanomaterials with photochromic moieties is a widely exploited approach to confer them the capacity to respond to light stimuli at specific wavelengths. The photochromic system's isomers possess markedly different properties at the single molecule level which give rise to remarkably diverse macroscopic properties, that is, at the ensemble level^27^,^28^,^29^,^30^. Therefore, the decoration of carbon-based nanomaterials with photochromic molecules to control local variation in the optical, mechanical, electrostatic environment via a light input has sparked great interest in the community of chemistry, physics and materials science. The combination of photochromic molecules with carbon-based nanomaterials is therefore being pursued to develop bi- or multi-functional molecular materials: the hybrid system will not only possess the unique properties of each component, but shall also feature the emergence of new properties that can potentially be used for specific applications. In this review, we provide a critical literature survey on the most enlightening results reported on carbon-based nanomaterials functionalized with photochromic molecules with a particular focus on the functionalization methods, on the dynamic properties conferred to the materials and their applications when integrated in working devices. The potential of these smart materials for the generation of novel, multifunctional, low-cost, large-area and flexible devices is discussed.

## Functionalization strategies

**Figure 2:**
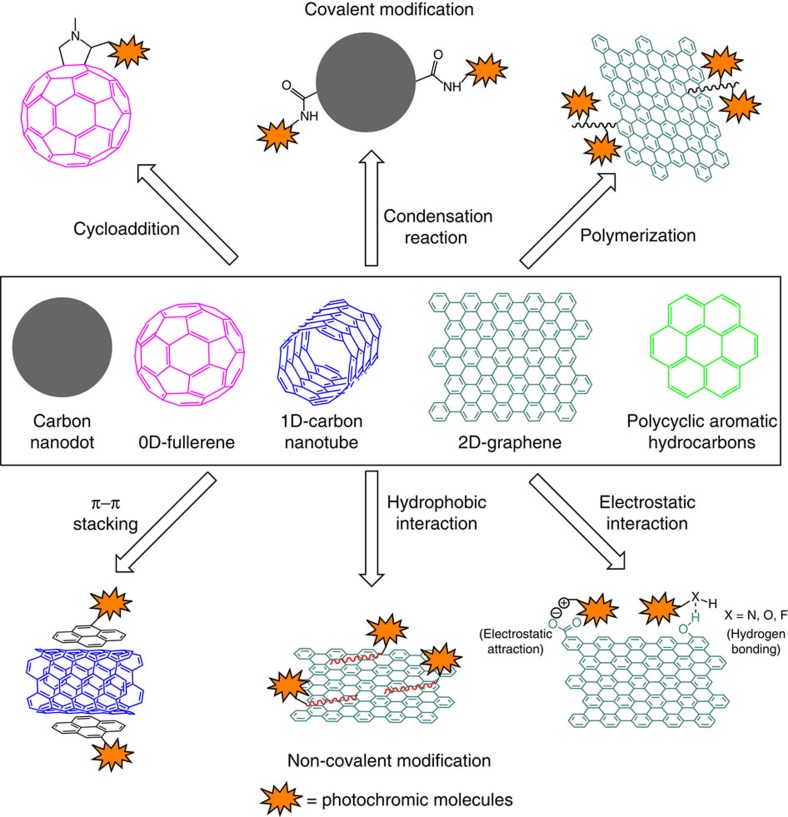
Functionalization of carbon-based nanomaterials with photochromic molecules. The functionalization can be performed through either covalent or non-covalent approaches. Non-covalent modification includes π–π stacking, hydrophobic interaction or electrostatic interaction, which only mildly perturbs the sp^2^ structure of the carbon allotrope. While covalent functionalization can be done via cycloaddtion, condensation reaction or radical polymerization and so on, offering strong and robust bonding. 0D, zero dimensional; 1D, one dimensional; 2D, two dimensional.

Functionalization of carbon-based nanomaterials such as fullerene, carbon nanodots, CNTs, graphene (including large polycyclic aromatic hydrocarbons) with molecular moieties possessing suitable properties is essential for the realization of new molecular hybrid materials with novel/enhanced functions. Regiospecific substitutions of carbon-based nanomaterials with functional groups in the scaffold and/or in the periphery is a viable approach to improve their stability, dispersibility thus processiblility, and provide tools to introduce new functions that can be used to further tune their properties. Functionalization of carbon-based nanomaterials with photochromic molecules including azobenzenes, diarylethenes, spiropyrans/-oxazines, and stilbenes can be achieved either through non-covalent^31^,^32^,^33^,^34^,^35^,^36^,^37^,^38^,^39^,^40^,^41^,^42^,^43^,^44^,^45^,^46^ or covalent approaches^47^,^48^,^49^,^50^,^51^,^52^,^53^,^54^,^55^,^56^,^57^,^58^,^59^,^60^,^61^,^62^,^63^,^64^,^65^,^66^,^67^,^68^,^69^. On the one hand, non-covalent modification of carbon-based nanomaterials is typically based on physical adsorption of photochromic units on carbon nanomaterials via π–π stacking, hydrophobic interaction or electrostatic interaction; such a strategy is advantageous since it only mildly perturbs the sp^2^ structure of the carbon allotrope, thus it is a viable method to adjust the properties of carbon-based nanomaterials via electronic communications. Because of these reasons, non-covalent functionalization has been widely used to build carbon-based functional optoelectronic devices. Pyrene unit is being frequently employed as an anchoring group for non-covalent functionalization of carbon-based nanomaterials with photochromic molecules^35^,^36^,^37^,^38^,^39^,^40^. On the other hand, covalent functionalization between carbon-based nanomaterials and photochromic molecules via condensation reactions (amidation^48^,^49^,^50^,^51^,^52^,^53^,^54^,^55^,^56^ or ester formation^57^,^58^,^59^), cycloaddition^60^,^61^,^62^, click chemistry^63^, diazonium chemistry^64^,^65^ and radical polymerization^66^,^67^,^68^ and so on^69^, enables strong and robust bonding and can offer a control over the degree of functionalization (Fig. 2). In terms of covalent functionalization, one common and convenient approach is to oxidize carbon atoms to generate carboxylic groups, which can be subjected to further functionalization via condensation reactions^48^,^49^,^50^,^51^,^52^,^53^,^54^,^55^,^56^,^57^,^58^,^59^. The degree of functionalization of carbon-based nanomaterials with photochromic molecules can be determined using thermal gravimetric analysis and X-ray photoelectron spectroscopy. It reveals that for every dozens to several hundreds of carbon atoms in the carbon-based core, there is one photochromic moiety appended, depending on the chemistry and conditions employed^51^,^53^.

## Properties of photochromic carbon-based nanomaterials

Due to the responsive nature of photochromic molecules, light can be used as an external remote control to reversibly regulate the properties of carbon-based materials and devices. Changes at the single-molecule level such as geometry/conformation, electronic structure, dipole moment, redox potential, can result in remarkably diverse properties at the materials level including shape^70^, aggregation behaviour^34^, conductance^21^, magnetism^71^, fluorescence^55^,^68^ and so on^72^. Examples of modulation the properties of photochromic carbon-based nanomaterials are summarized in Table 2. The modulation of the properties not only depends on the photochromic molecules and carbon-based materials employed, but it is also affected by the interactions between the two components, the measurement conditions and so on. For example, modulation of the properties of graphene using photochromic molecules depends on the type of graphene used (reduced graphene oxide (rGO) versus mechanical exfoliated graphene versus graphene grown by chemical vapour deposition); there is a dramatic difference in the modulation range of dipole moment between using azobenzenes and spiropyrans, which can affect the charge transport properties in the hybrid materials. Compared with alkyl substituted photochromic molecules, pyrene-substituted ones interact differently with carbon-based nanomaterials, which will affect the experimental results; the distance between photochromic molecules and carbon-based nanomaterials also plays an important role in the interactions. Other factors like irradiation time, solvents used or level of vacuum also contribute to the final performance of the hybrid systems.

Among the various properties, a subtle control over the energy level alignments of the different components/moieties enables the use of light to control electronic cross-talking between different units in a multicomponent material leading to the modulation of fundamental optoelectronic processes such as charge generation, transfer and recombination as well as energy transfer^73^,^74^,^75^,^76^,^77^,^78^, which are ruling the operation of light-emitting diodes, organic solar cells, artificial photosynthetic systems and single-molecule devices. The type of chosen photochromic molecules and carbon-based nanomaterials as well as the chemical nature of the linker will define the charge transfer/separation processes between them, thereby influencing the efficiency of the photochromic system (for example, time response to the external stimuli, lifetime of the isomers, fatigue resistance and so on). For example, photo-induced geometrical/structural changes of photochromic molecules or changes of the length/structure of the linkers can be used to regulate the distance/conjugation between the two components, thus can modulate the efficiency of electron and/or energy transfer processes. On the other hand, the widespread use of fluorescence together with the need to further understand how to design and control the photophysical properties of functional (nano)materials have stimulated the identification of mechanisms to reversibly switch the emission of these (nano)materials with external stimuli^31^. In particular, strategies to reversibly modulate the fluorescence of carbon-based nanomaterials using light have already been developed successfully based on electron and/or energy transfer processes^55^,^68^, which might lead to novel photonic materials for information technology as well as luminescent probes for biological applications. The packing and orientation of photochromic molecules in carbon-based nanomaterials can be also altered by light, resulting in changes in conformation/structure to be used as molecular machines, actuators or sensors. By controlling the irradiation time, intensity and spot location, light can be very easily utilized to tune the degree of modification of the target function to realize multiscale responsive carbon-based nanomaterials controlled both in time and space, for example, as a viable approach to fabricate multilevel non-volatile optical memories.

## Applications of photochromic carbon-based materials

The ability to modulate the functions of (nano)devices using external stimuli is highly desirable in microelectronics, nanoelectronics and especially in optoelectronics due to their potential applications in switching, sensing, memories and so on. In view of their outstanding light responsive nature, photochromic carbon-based nanomaterials are particularly suitable for these broad applications. Here we discuss recent progresses on the integration of photochromic carbon-based nanomaterials as components in nanodevices (for example, as molecular junctions^79^,^80^,^81^,^82^,^83^,^84^,^85^,^86^,^87^,^88^, as electroactive-species in field-effect transistors (FETs)^89^,^90^,^91^,^92^,^93^,^94^,^95^,^96^,^97^,^98^), and optoelectronic devices (for example, optical read-out^99^, optical memories^42^, color detector^100^, and solar thermal fuels^20^). Alongside, their application in sensing^100^, including biomedical imaging and drug delivery^52^ will be also highlighted. Photochromic molecules whose isomers exhibit remarkably different properties, when combined with carbon-based nanomaterials, are also potentially useful to fabricate smart/dynamic (nano)materials (for example, smart nanocomposites/windows/labels)^27^. Towards multifunctional materials, in addition to the integration of photochromic systems that can respond to a light input, carbon-based nanomaterials can be decorated with functional groups that respond to other stimuli like pH and/or electric fields. The read-out can occur optically or electrically.

## Molecular junctions

The electrical characteristics of photochromic molecules can be explored by embedding them in-between two electrodes in a molecular junction. Molecular junctions rely on nanoscopic or macroscopic electrodes whose geometry and spacing can enable the measurement of the transport through single molecules. Because of its fundamental quantum-mechanical nature, charge transport through molecular junctions is an exciting field, at the cross-road between mesoscopic physics, quantum computing and spintronics.

Being the switch, a key element in (nano)electronics, the ability of gating the conductance of a single molecule using an external remote control has a paramount importance. The electrical characteristic of an electrode–molecule–electrode junction is governed by the structure and energetics of the two components and their interfaces, as well as by the ability of the chosen molecule to transport charges. The former requires to achieve a high-structural control over the electrode surface and its combination with functional groups attached in the periphery of the molecule to guarantee precise (non)covalent linkage^79^. Compared with other electrode materials like metals, carbon-based materials possess obvious advantages, such as extraordinary and modulable electronic properties, natural compatibility with molecules to enable more stable contacts. Bridging carbon-based electrodes with photochromic molecules is a viable route towards conferring an optical response to an electronic device.

Cuniberti and co-workers^21^ reported a theoretical investigation on the charge transport of a prototypical junction consisting of single azobenzene molecule connecting two CNT electrodes. They showed that the low energy conduction properties of the junction may be drastically modified by changing the topology of the contacts between the CNTs and the photochromic molecule, and/or the chirality of the CNTs. Later on, several theoretical studies on the combination of photochromic molecules with CNTs/graphene have been carried out^80^,^81^. The conductivities were found to be significantly different for the two photochromic isomers. All these theoretical studies have triggered an experimental effort and contributed to the understanding of the experimental results.

**Figure 3:**
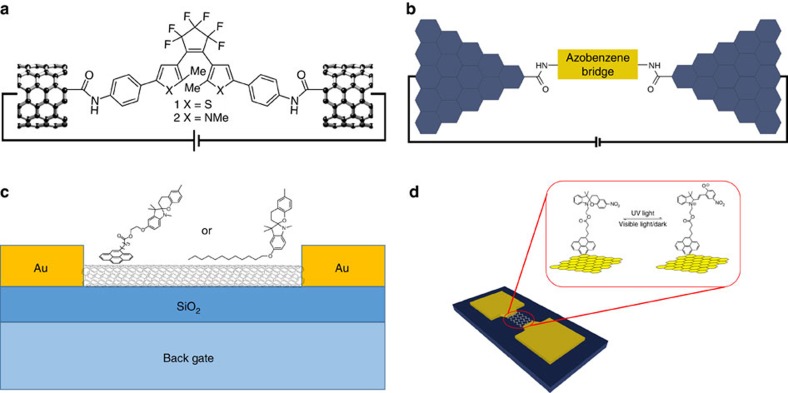
Applications of photochromic carbon-based nanomaterials in molecular junctions (upper images) and transistors (lower images). Amide formation is used (**a**) to bridge two CNTs with a diarylethene molecule. Adapted from ref. 82 (Copyright 2007 American Chemical Society) and (**b**) to connect covalently two graphene point contacts with an azobenzene molecule. Adapted from ref. 84 (Copyright 2013 John Wiley & Sons, Ltd.). (**c**) Spiropyrans derivatized with either alkane or pyrene groups were physisorbed on CNTs. Adapted from ref. 89 (Copyright 2005 American Chemical Society). (**d**) A graphene-based field-effect transistor functionalized with pyrene-substituted spiropyrans. Adapted from ref. 94 (Copyright 2012 American Chemical Society).

Contacting a single molecule with two symmetric electrodes is not a trivial task. Importantly, the spacing between adjacent electrodes needs to match exactly the size of the molecule. Nuckolls and co-workers^82^ reported an experimental work that relied on the bridging of two CNTs with a diarylethene molecule via amide formation. The CNTs point contacts were made by electron beam lithography. In their system, two different diaryethene molecules were used (Fig. 3a). When X=S, the thiophene-based devices show only one-way switching from open to closed state, changing from a lower conductance state to a higher conductance state. When X=NMe, the pyrrole-based devices can be switched on/off for several times. They hypothesized that the CNT electrode dissipates the energy generated in the excited-state of the closed form of thiophene-based diarylethene (X=S). These results highlight the importance of molecular structures, which drastically affect the device characteristics and its final performance. If the electronic coupling between the electrode and the photochromic molecule is sufficiently strong, on irradiation with light, either electron or hole may leak out to the electrodes on a timescale shorter than the time needed for the photochrome to switch, which will result in the loss in the photochromic functionality.

Single-molecule junctions based on the wiring of graphene electrodes with diarylethene have also been reported^83^. Graphene point contacts arrays with narrow gaps were patterned by a dashed-line lithography process with carboxylic acid terminal groups, which can react with diarylethene bridges derivatized with amino moieties via amide tethering. Under ultraviolet light irradiation, the devices show a photo-switching behaviour from low-conductance state to high-conductance state, which is due to the ring closing of the diarylethene generating a conjugated pathway for charges travelling from one graphene electrode to the other. However, as a result of the quenching induced by the strong coupling between diaryethenes and graphene electrodes, the devices cannot isomerize back to the low-conductance state. By using a similar approach, the same group was able to achieve a reversible conductance switching of azobenzene units covalently connecting to two graphene point contacts on device exposure to external light (Fig. 3b)^84^. Owing to the presence of two sulfonic acid groups in the azobenzene molecule, the conductance of the device is also sensitive to pH stimuli. It is worth mentioning that the conductance changed more than two orders of magnitude from acid to basic condition, thus providing an ultrasensitive local probe for monitoring pH changes based on single molecules. This study represents a good example of designing multifunctional single molecular devices towards the construction of logic gates or even molecular computers. Although the above symmetric junctions consisting of two CNTs or graphene-based electrodes showed promising results, bridging the interelectrodic gap with an atomic precision using single molecules remains a major challenge.

Vertical junctions incorporating photochromic molecules have been also realized and characterized. A photo-switchable self-assembled molecular monolayer (SAM) of thiol-substituted dihydroazulenes has been sandwiched between a gold electrode and an ultrathin thermally rGO film acting as transparent top contact, allowing *in situ* illumination to attain photo switching; optical–thermal stimuli was therefore employed to run the device^85^. Reversible conformational change between dihydroazulenes and vinylheptafulvene was triggered by alternating light irradiation and heat treatment, with an average *I*_on_/*I*_off_ ratio of 5–7. This *I*_on_/*I*_off_ ratio is smaller than the one determined in the molecular junction incorporating photoactive azobenene or diarylethene^82^,^84^,^87^. In this case, rGO was chosen because of its high transparency, good conductivity and flexibility, solution processability, excellent chemical stability and molecular compatibility. Lee and colleagues^86^ independently developed a method to fabricate flexible and photo-switchable vertical junctions using graphene as electrodes. In their approach, azobenzenes were covalently attached to the graphene bottom electrode via diazonium chemistry so as to prevent the local movement of photo-switchable molecules, while the other side of the azobenzenes is physically contacted to the graphene top electrode. The graphene electrodes were prepared by a chemical vapour deposition method, which yields higher conductivity compared with rGO films. The as-fabricated devices are stable under severe mechanical stress and for a large number of photo-switching cycles with an *I*_on_/*I*_off_ ratio ∼13, suggesting the potential application in flexible switching devices.

More recently, a graphene/azobenzene/Au heterostructure was designed by Margapoti *et al*.^87^. In this case, a mixed SAM of an azobenzene derivative and a spacer molecule has been co-adsorbed, the latter component allowing to prevent steric hindrance of the photo-induced conformational switching and intermolecular coupling of the azobenzene. The strength of the current resonances determined by conductive atomic force microscopy can be reversibly gated optically with an *I*_on_/*I*_off_ ratio exceeding 100; such a switch was found to further induce the reversible modification of the electrical and quantum properties of the Dirac fermions of graphene. In a previous report by our group together with Mayor^101^, a conductive atomic force microscopy tip/azobenzene/Au junction was devised, without the use of graphene and a spacer molecule, and it revealed a 30-fold increase in conductance as a result of the conformational change of azobenzene in a process ruled by tunnelling. The change in conductance is smaller than the one reported for graphene/azobenzene/Au junctions. Devices with a rGO/diarylethene/Au heterostructure on a flexible substrate have also been fabricated^88^, showing a reversible conductance change in response to alternating irradiation with ultraviolet/visible light. In addition, the switchable devices show good longtime stability under various mechanical deformations.

Compared with CNTs, an obvious advantage of graphene as an electrode material lies in the fact that it does not have the inherently variable diameter and chirality, which should rule out the possibility of modulating the junction conductance by the electrode chirality as it has happened in CNTs. Compared with electrodes using rGO and graphene prepared via chemical vapour deposition, the advantage of the former lies in its solution processability while the latter retains the intrinsic exceptional properties of graphene.

## Field-effect transistors

While molecular junctions are essential to gain insight into the possibility of transporting charges through single molecules, their technological relevance is limited because of the excessive costs associated to the scaling down lithographic procedures. However, the information gathered on the charge transport through single molecules is instrumental towards the development of high-performance macroscopic devices. In this regard, FETs, which are the key element of microelectronics, are mono-functional devices that rely on the electrical gating to modulate transport of one type of charge carrier in a semiconducting material. The integration of a photo-responsive molecule in a transistor allows to develop a bi-functional device, that is, a device which is capable of responding to two independent stimuli. Because of this reason one of the most worthwhile application of photochromic carbon-based nanomaterials is their use as electroactive-species in FETs. One practical way to combine electrically (semi)conducting materials with photochromic systems is by means of non-covalent interactions. The functionalization of CNTs with spiropyrans was demonstrated by Nuckolls and co-workers^89^. In their study, spiropyrans derivatized with either alkane or pyrene groups were physisorbed on CNTs (Fig. 3c). For spiropyrans, the charge-separated ring-opened, coloured form named merocyanine is generated by irradiation with ultraviolet light, whereas the reversing ring closure is triggered with visible light to form the neutral, colourless form. Such a reversible photo-isomerization process causes a significant change in the dipole moment, which could initiate a significant modification in the electrostatic environment. FETs based on these spiropyran functionalized CNTs could be switched from high to low-conductance state as the spiropyrans photo-isomerized from the open to the closed state. The merocyanines acted as scattering sites and/or charge traps, leading to a decrease in conductance. These devices can detect the photo-switching events of ∼10^4^ molecules. This study represents a good example of combining microfabrication with self-assembly of photo-responsive molecules towards functional FETs. Using a similar approach, azobenzenes with an anthracene anchoring group were physisorbed on CNTs^90^. When azobenzene undergo photo-isomerization from the *Z* to the *E* form, the resulting change in dipole moment modifies the local electrostatic potential and modulates the transistor conductance by shifting the threshold voltage. A shift in the threshold voltage of up to 1.2 V was observed at a degree of functionalization of ∼1–2 azobenzene molecules every 100 carbon atoms. Importantly, the conductance change was reversible and repeatable for a long period of time, with a modest of ∼2 s switching time.

Similar strategies have also been applied to realize graphene-based transistors. In this framework, Gopalan and co-workers^91^ reported light-driven reversible doping of graphene by pyrene-substituted azobenzenes. The charge carrier concentration could be modulated by ∼±1 × 10^12^ cm^−2^ by ultraviolet and white light illumination, meanwhile preserving charge carrier mobilities of pristine graphene. Compared with substitutional doping, the retention of high mobilities, the ability to reversibly modulate doping using light and the ease of processing are clear advantages of this study^91^. Peimyoo *et al*. studied the doping of azobenzenes without pyrene groups directly deposited on graphene^92^. The *E* form modified graphene shows stronger doping effect than that of *Z* form due to the molecular conformation change (charge carrier concentration from ∼5 × 10^13^ to ∼4 × 10^13^ cm^−2^), which has different interactions with graphene. Using a similar strategy, our group together with M. Mayor reported the modification of the source and drain Au electrodes of an organic FET with a thiolated biphenyl azobenzene SAM^102^. The semiconductor was located in the transistor channel. The current in the as-fabricated device can be reversibly modulated by 20%, which is attributed to the photochemical modulation of the tunnelling barrier for injection/extraction of charges from the electrodes into the organic semiconductor. Conversely, in the above examples of azobenzene modified carbon-based FETs, the azobenzene molecules were directly deposited on the top of CNT/graphene^90^,^92^,^93^.

In a later report, reversible light-modulated doping of graphene using pyrene-substituted spiropyrans was also described^94^,^95^. Changes in Dirac point and mobility are reversible via alternating ultraviolet and visible light irradiation, and the doping level of graphene functionalized with spiropyrans can be controlled. The hole and electron mobility of the functionalized graphene with spiropyrans after ultraviolet irradiation slightly decreased to 508.8 to 392.4 cm^2^ V^−1^ s^−1^, and from 428.4 to 301.2 cm^2^ V^−1^ s^−1^, respectively (Fig. 3d)^94^, a result that has been attributed to charge scattering. The electrical conductivity of a photochromic blend composed of polymers having diarylethene derivatives in the main chain and CNTs as active components in transistors is reversibly tuned on cycling ultraviolet and visible light^96^. The photo-switching capacities of the diarylethene in the main chain of the polymer observed in solution are retained on physisorption on the CNT surface. Based on electrical and spectroscopic evidence, the authors concluded that the intertube electrical coupling, mediated by the light-induced electrocyclization of the switch, is responsible for the conductivity modulation of the device.

As alternative strategy to the above non-covalent functionalization approach, azobenzenes were covalently tethered onto chemically rGO sheets, whose carrier density can be modulated reversibly via light irradiation^97^. A change in dipole moment from *E* to *Z* leads to the generation of a high effective gating voltage. All these examples of ‘light-modulated' transistors open up new pathways towards the generation of optical interconnects and light-addressable logic circuits.

## Solar thermal storage

**Figure 4:**
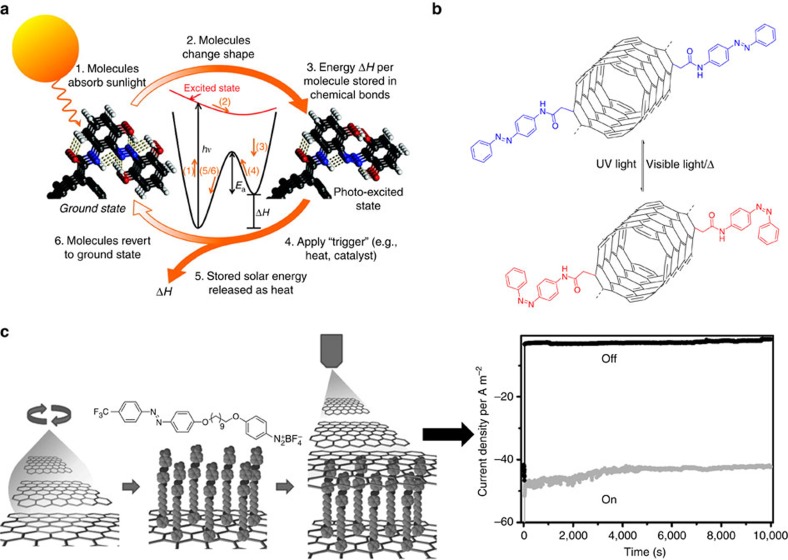
Applications of photochromic carbon-based nanomaterials in solar thermal storage and memory devices. (**a**) Mechanism of solar thermal fuels based on azobenzene covalently linked to CNTs. Reproduced from ref. 104 (Copyright 2011 American Chemical Society). (**b**) Scheme for photochemical and photochemical/thermal cycling of azobenzene covalently attached to CNTs used for solar thermal fuels. Figure 4b is drawn according to ref. 20 (Copyright 2014 Nature Publishing Group). (**c**) Voltage-controlled non-volatile molecular memory devices by using an azobenzene monolayer as the active layer sandwiched between two rGO electrodes via a solution process, and memory-retention performances of the ON state and the OFF state. Reproduced from ref. 108 (Copyright 2013 John Wiley & Sons, Ltd.).

Solar energy conversion and storage are one of the key challenges in modern society. Among them, effective conversion of light into heat is an emerging area showing great potential for a number of applications, such as in solar thermal storage. Solar thermal fuels can store energy from light in photochromic molecule's isomerization process, with their metastable forms capable of releasing nearly 100% of the stored energy as heat as the result of external stimuli along with the reversion to the stable forms. Solar thermal fuels are regarded as a closed-cycle system that only transfers energy from and to the environment by reversible transformation^20^. However, solar thermal storage is still hindered by a low storage capacity, short-time storage and lack of novel designed structures. Recently, novel hybrid solar thermal fuels have been prepared through covalent linkage of CNTs with azobenzenes, as demonstrated both theoretically and experimentally by Grossman and co-workers^20^,^103^,^104^. Azobenzene undergoes a conformational change under ultraviolet light irradiation from *E* to a higher energy metastable *Z* form, thus storing energy Δ*H*. To recover the stored energy, an external trigger is applied to overcome the thermal barrier *E*_a_, thus releasing a net energy of Δ*H* in the form of heat per azobenzene molecule (Fig. 4a). In the system, CNTs not only acts a template for packing and orientation of the tethered azobenzene molecules, but also have a significant effect on the energetics of the system by breaking the symmetry of the azobenzene molecules. A relatively low energy density of 56 Wh kg^−1^ was obtained due to the low packing densities of azobenzene attached onto CNTs through radical chemistry (one azobenzene every 18.2 carbon atoms, see Fig. 4b for the chemical structure of the hybrid)^20^. The condensation of the hybrid materials in the solid-state by solvent removal to form a more close-packed arrangement led to twofold enhancement of the storage energy capacity. The selection of the carbon-based templates and the orientation of the photochromic molecules can also affect the final performance of the devices. Using a different chemical approach, azobenzenes were also chemically attached onto rGO via diazonium chemistry for long-term solar thermal storage by Feng's group^105^,^106^,^107^. Optimizing intermolecular hydrogen bonding interactions and also increasing the functionalization degree (up to one azobenzene every 17 carbon atoms) can remarkably improve both the storage capacity and lifetime, showing an energy density up to 138 Wh kg^−1^, a long-term storage half-life exceeding 1 month and excellent cycle stability for 50 cycles^107^. However, the packing density of azobenzene in the above hybrid systems is still far beyond the optimized value predicted by theory (i.e., one azobenzene every eight carbon atoms)^103^,^104^, suggesting that new chemical approaches need to be devised to improve the degree of functionalization in carbon-based templates using photochromic molecules.

## Memory devices

With the development of information technology, there is a high demand to exploit new methods for data storage. In this framework, the maximization of the data storage densities is a major goal in memory devices. This can be achieved by developing all-optical switching devices that exhibit high switching rates or multilevel storage. An ideal memory device should be stable, easy to write, read and erasable. Photochromic molecules that change their state reversibly in response to external stimuli could be used to build non-volatile memory devices: their data storage capacity allows writing and erasing data in photon mode with a high spatial and temporal resolution. Carbon-based nanomaterials, and especially graphene, are excellent candidates as electrodes in memory devices due to their unique properties such as high conductivity, transparency and good flexibility. Memory devices consisting of photochromic molecules as an active layer sandwiched between two graphene electrodes have been developed. As a typical example, Min *et al*.^108^ reported the fabrication of voltage-controlled non-volatile molecular memory devices by using an azobenzene monolayer as the active layer incorporated between two chemically rGO films acting as electrodes via an all-solution-processed approach (Fig. 4c). First, a graphene oxide film was spin-coated onto an indium tin oxide coated plastic substrate, followed by a hydrazine vapour reduction step to obtain the rGO film, which was then used as the bottom electrode. In the second step, a monolayer of azobenzene was chemically attached onto the rGO film via diazonium chemistry. Finally, a device with a configuration of rGO/azobenzene monolayer/rGO was obtained by spray-coating of rGO solution as the top electrode. The as-fabricated devices showed a typical rewritable memory behaviour with an *I*_on_/*I*_off_ ratio of 20 and a retention time exceeding 10^4^ s (Fig. 4c), which is due to the resistive switching of the azobenzene monolayer (while the control device incorporating only rGO did not show any memory effect). The non-volatile memory exhibited a stability exceeding 400 cycles of write–read–erase–read. Interestingly, these devices also showed good memory characteristics under bending stress, demonstrating their potential use in flexible electronic devices. However, the influence of the degree of reduction of rGO on the device performance is yet to be studied. Optical controlled graphene-based non-volatile ternary-logic memory with azobenzene copolymer as an active layer was also demonstrated^109^. In this case, mechanical cleavage graphene was used as the bottom electrode and indium tin oxide was employed as the top electrode. The graphene-based devices can perform ternary logic, and the resistance change ratio of the written status ‘±1' to the erased status ‘0' was 60%. Instead of using azobenzenes, diarylethenes would be a more suitable candidate for memories devices due to its bistable nature and excellent fatigue resistance. Furthermore, improving the reproducibility from device to device and a better understanding of the memory mechanism should be further explored.

## Sensing

Each constituting atom of CNTs and graphene is exposed to the environment. Because of this reason these nanostructures are extremely sensitive to environment modification at the nanoscale, thus they are ideal platforms for sensing. Photochromic carbon-based nanomaterials are a class of promising sensors due to the intrinsic isomerization and colorimetric/fluorescent properties. To date, sensitivity and selectivity of sensors based on photochromic carbon-based nanomaterials are major features that need to be harnessed. This may be achieved via the decoration of the building block with units that can selectively recognize the molecules to be sensed via specific interactions. Via rational design of photochromic carbon-based nanomaterials, it should be possible to achieve visual, sensitive and high-throughput detection.

**Figure 5:**
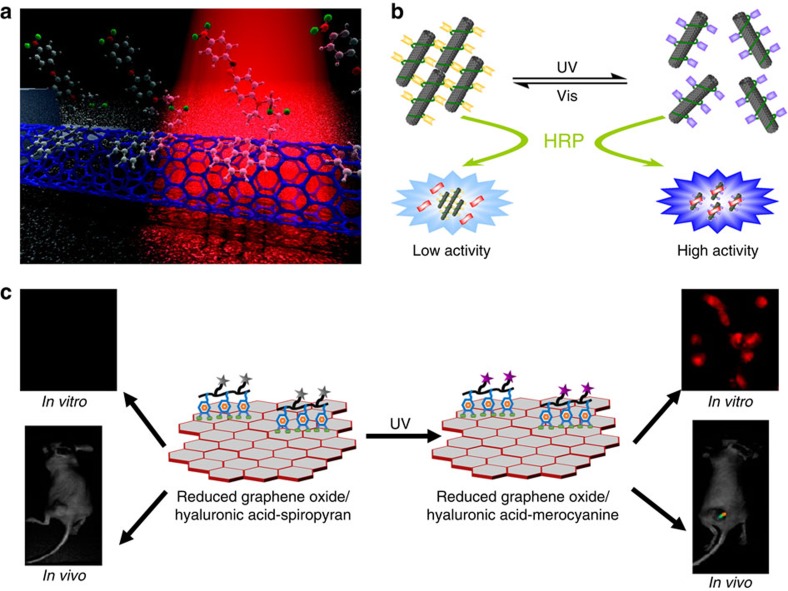
Applications of photochromic carbon-based nanomaterials in sensing (upper images) and biology (lower image). (**a**) CNTs modified with azobenzenes were used as a color detector. Reproduced from ref. 100 (Copyright 2009 American Chemical Society, http://pubs.acs.org/doi/pdf/10.1021/nl8032922). (**b**) Spiropyrans covalently attached to CNTs were used to regulate horseradish peroxidase (HRP) activity via light irradiation. Reproduced from ref. 110 (Copyright 2011 Royal Society of Chemistry). (**c**) rGO/hyaluronic acid-spiropyran used for *in vivo* fluorescence imaging. Adapted from ref. 114 (Copyright 2013 American Chemical Society).

Zhou *et al*.^100^ reported a nanoscale color detector based on CNTs modified with azobenzenes, where the azobenzenes serve as photo-absorbers and the nanotube as the electronic read-out (Fig. 5a). By synthesizing azobenzenes with specific absorption windows in the visible region and anchoring them to the nanotube surface via pyrene linker group, they demonstrated the controlled detection of visible light of low intensity in narrow ranges of wavelengths. The authors suggest that on photoexcitation, azobenzenes undergo isomerization from the *E* state to the *Z* state, accompanied by a large change in dipole moment, therefore, modifying the electrostatic environment of the nanotube. The calculated values of the dipole moments support the notion of dipole changes as the optical detection mechanism. This system can be used to study fundamental properties of chromophore–nanotube hybrids and to probe molecular transitions. This approach represents a useful way to make colour photodetectors with nanoscale resolution.

Qu and co-workers^110^ exploited the amidation reaction to chemically graft spiropyrans to CNTs, and used such a hybrid system to regulate horseradish peroxidase (HRP) activity via light irradiation (Fig. 5b). On ultraviolet light irradiation, the hybrid system showed enhanced catalytic HRP activity, which comes from the synergetic effect of CNTs and the merocyanine form of spiropyrans. The enhancement in the catalytic HRP activity has been used as a label-free colorimetric assay of lysozyme with a detection limit of 30 nM. The high selectivity is due to the specific binding between lysozyme and aptamer. This approach does not need to covalently attach photochromic molecules on proteins and can be applicable to regulate the activity of other natural proteins using light. In another report, Lee *et al*.^111^ prepared a nanocomposite material assembled from azobenzene functionalized graphene oxide and stilbene-metal organic frameworks, which is capable of luminescent quenching by explosive gases. This unique system displays selectivity to dinitrotoluene (71% quenching) over trinitrotoluene (20% quenching) with sub-p.p.m. sensitivity and response times of less than a minute. This study opens a route towards the design molecularly well-defined materials possessing rapid, reversible and gas-selective fluorescent quenching capabilities. Yang *et al*.^112^ designed a nanocomposite consisting of a silyl-appended spiropyran and graphene oxide, which has been used as a chemosensor for the rapidly, selectively and sensitively colourimetric detection of fluoride ions in aqueous solution based on a specific chemical reaction. A nucleophilic reaction between silyl-appended spiropyran and fluoride ions promoted the ring-opening process, thus leading to increased absorption and colour change from colourless to orange-yellow. Graphene oxide could shorten the response time of silyl-appended spiropyran to 1/6 and lower the detection limit more than one order of magnitude, due to the large specific surface area and hydrophilic groups on graphene oxide improving the solubility of the silyl-appended spiropyran. The fast, selective and accurate determination of fluoride ions holds great potential in both environmental and biological systems. Using a similar strategy, they further extended the nanocomposite system in bioimaging of fluoride ions due to the nanocarrier function of graphene oxide for cells^113^. This approach will promote the further exploration of more effective fluorescent nanodosimeters for other analytes. However, novel photochromic carbon-based nanomaterials enabling multi-analyte detection via the observation of different signal outputs should be designed with the ultimate goal of discriminating analytes having similar structure/property, towards the fabrication of an artificial nose.

## Biological applications

The ability to modulate structure and function in response to energy input and external signals is an important aspect also when implemented in biological systems, with the goal of realizing reversible switching of biological function between two different states (for example, inactive and active). Photochromic molecules have been used to modulate a number of important biological processes, such as protein folding, enzyme activity, membrane transport and so on. On the other hand, carbon-based nanomaterials are also widely employed for biomedical imaging, drug delivery and cancer therapy. Photochromic carbon-based nanomaterials capable of responding to a light input already showed to be active building blocks for biological applications, such as fluorescent probe for diagnosis and drug carrier in drug delivery system. For these purposes, photochromic carbon-based nanomaterials possessing a fast switching process and a large extinction coefficient with a high quantum yield is required. Del Canto *et al*. used amide coupling to link spiropyrans to CNTs, which showed light-modulated release of antinflammatory zinc ions^52^. The realization of potential photo-controllable spiropyran/CNTs based drug delivery systems is envisaged, where CNTs act as intracellular carriers of light-modulated receptors for bioactive agents. Park and co-workers^114^ demonstrated a strategy to prepare photo-responsive chemically rGO with mussel-inspired adhesive material dopamine and photochromic dye spiropyran conjugated to the backbone of the targeting ligand hyaluronic acid. rGO/hyaluronic acid-spiropyran could retain *in vivo* fluorescence imaging feature using confocal laser scanning microscope (Fig. 5c). Accumulation of rGO/hyaluronic acid-spiropyran in tumour tissue from biodistribution analysis strongly supports the specific delivery of prepared graphene to the target destination. Later on, the same group reported a stimuli-responsive fluorescent nanomaterial based on graphene oxide coupled with a polymer conjugated with photochromic spiropyran dye and hydrophobic boron dipyrromethane dye, for application in light-triggered target multicolour bioimaging^115^. The stability, biocompatibility and quenching efficacy of this nanocomposite open perspectives for cell imaging in different independent colours, sequentially and simultaneously. Although the above results are promising, the risk of using carbon-based materials in biological systems should be always considered, and designing photochromic molecules capable of switching with infrared light irradiation still need to be solved for the practical applications.

## Other applications

Photochromic carbon-based nanomaterials hold potential for applications in other emerging fields. For example, the use of azobenzenes blended with CNTs and polymers to form nanocomposites possessing light-induced conductance switching properties have been reported^116^,^117^. These nanocomposites appear as good candidates for electro-optical memories, smart windows or ‘smart labels' in packaging, in particular anti-counterfeit, applications.

Chemically rGO was functionalized with azobenzene-polymer brushes via surface initiated controlled radical polymerization, which was further used for photo-induced surface relief gratings^67^. Experimental results showed that light can be used to induce transport of graphene sheets within the molecular glass matrix on the micrometre scale. Mechanically cleavaged multilayer graphene was exploited as a nanoscopic probe to characterize local opto-mechanical forces generated within photosensitive polymer films containing azobenzene units^118^. On irradiation with light interference patterns, photosensitive films deform according to the spatial intensity variation, leading to the formation of periodic topographies such as surface relief gratings with internal pressure exceeding 1 GPa. This study opens the possibility to characterize opto-mechanical forces generated within photo-responsive polymer films.

Unlike macroscopic motion in real-world systems, molecular systems are constantly undergoing Brownian motions. Because of this reason harnessing molecular motion unidirectionally is a far more difficult challenge. Nanovehicles are one kind of molecular machines having a chassis connected to wheel-terminated axles, which can convert energy inputs into motion on a surface in a controlled manner, by bridging the gap between synthetic molecular systems and the machines of the macroscopic world. From the synthetic point of view, synthesis of nanovehicles itself from ‘bottom-up' using molecular building blocks is quite demanding and challenging. Photochromic carbon-based nanomaterials based on fullerenes are used for the design of nanovehicles, by taking advantage of the remarkably different dipole moments and nanoscale mechanics of the photochromic isomers and the perfect spherical structure of fullerene. Tour and co-workers^119^ designed a branched azobenzene-fullerene-based nanostructure to realize a nanovehicle aiming to move like a caterpillar. Despite the designed nanovehicle did not show any movement on surfaces, this seminal work represents a source of inspiration for future design of nanovehicles containing photochromic carbon-based nanomaterials. Nowadays a major challenge remains the synthesis of new vehicles capable of travelling over long distances with an atomic precision in the motion at surfaces and an exquisite directional control.

Highly ordered fullerene arrays were constructed at the liquid/solid interface by co-adsorption of fullerenes and a supramolecular template using an azobenzene derivative^120^. Other systems combining large polycyclic aromatic hydrocarbons with photochromic molecules self-assembled at the liquid/solid interface were also reported^121^,^122^. These studies provide new insights on how to control dynamic self-assembly on surfaces. Self-assembled graphene/azo polyelectrolyte multilayer film held potential use in electrochemical energy storage devices^123^. Interestingly, three-dimensional polyphenylene dendrimers combined with azobenzenes were used as light-driven encapsulation of guest molecules into interior cavities, which is important for the design of new drug delivery systems^124^. As a follow-up of this work, Müllen and colleagues^125^ further designed azobenzene–polyphenylene dendrimers with a borate anions core, which showed a reversible change in the conductivity of electrolyte solutions of the salt on light irradiation. This is due to photo-induced changes in the overall size of the dendronized anion and the density of its polyphenylene shell. A molecular ‘hexad' consists of five bis(phenylethynyl)anthracene fluorophores, a dithienylethene photochrome and a central hexaphenylbenzene unit has been synthesized and used as a photochemical ‘triode' molecular signal transducer^126^. Remarkably, multiple modulations such as fluorescence, frequency, amplitude and phase can be realized. However, the synthesis of the three molecules is challenging^124^,^125^,^126^, which may hinder their practical applications. All of these studies have largely broadened the application scope of photochromic carbon-based nanomaterials.

## Future perspectives

The progress of photochromic carbon-based nanomaterials meets the growing requirement for environmental friendly and smart materials, largely depending on advanced material design and characterization techniques^86^. Reduced response time combined with enhanced light sensitivity and electronic modulation of photochromic carbon-based materials will be key for the development of novel multifunctional responsive materials, for example, smart windows, new type of photodetectors, ultra-light weight photonic and electronic devices, flexible sensors and so on. When combined with cheap processing method, such as inkjet printing, spray-coating or roll-to-roll, photochromic carbon-based nanomaterials can be expected to have a bright future towards low-cost, large-area and scale production. Photochromic carbon-based nanomaterials can be also applied further in biology by tethering them to biomolecules: the ability of sensing conformational changes can be used to detect modification of biological environments or biological based recognition events leading to conformation changes like protein folding.

Despite the rapid progress, the field of photochromic carbon-based nanomaterials is still not fully ripened. What are the bottlenecks in the development of photochromic carbon-based nanomaterials? Several challenges can be envisaged and should be addressed in the future development of this field. Further fundamental insights are required through a deeper investigation of the working principles for photochromic molecules tethered with carbon-based nanomaterials, in particular by unravelling the relationship between structures at the molecular/supramolecular level and the photo-responsive behaviour. Photophysical and photochemical processes of photochromic carbon-based nanomaterials are still far from being fully unveiled (for example, how are the charges spatially distributed on excitation and how is the photo-isomerization process affected by the interaction between photochromic molecules and carbon-based nanomaterials), being a colossal task that can be accomplished by combining spectroscopic investigations with modelling/simulation efforts. New and advanced characterization techniques are needed to bridge theories and experimental results. Optimization of the efficiency for light responsiveness and enhancement of the long-term photostability of the hybrid system should be also considered as important aspects for applications.

**Figure 6:**
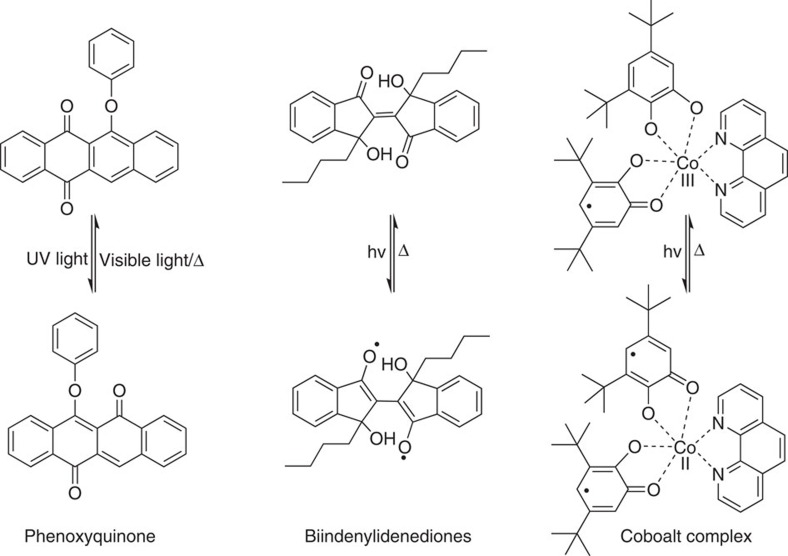
Potential interesting photochromic molecules. These molecules have not been combined so far with carbon-based nanomaterials, which can be used for specific applications. Intramolecular transfer of phenoxyquinone for sensing (ref. 130). Photo-induced radical formation of biindenylidenediones (ref. 131) or photo-induced valence tautomerization of cobalt complexes (ref. 132) for spintronics. The three examples proposed represent basic chemical structures, which can be possibly redesigned with different substituents.

For solar thermal fuels to become feasible, photochromic carbon-based nanomaterials with a much greater packing density of active photochromic molecules are required such that the energy density can approach that of commercial batteries^127^. For example, the high surface strain of fullerenes may allow a much higher density of photochromic molecules to be attached. Besides azobenzene, other photochromic molecules like stilbene derivatives could be also considered for incorporation in carbon-based solar thermal fuels^128^. Another possibility to improve the performance of solar thermal fuels is to introduce bulky functional groups on the carbon-based templates, thus increasing the rigidity of the photochromic molecules. As for the application in molecular junctions, better control of the interfacial electronic coupling between carbon electrodes and photochromic molecules by rational design of the molecular backbone of the latter is needed. For instance, increasing the flexibility of the junction either by avoiding conjugated bonds instead using longer saturated carbon linkers/metaphenyl or by enlarging the electrode mobility. For memory devices, a high *I*_on_/*I*_off_ current ratio, long-term stability, excellent reversibility and reproducibility, further increasing the rates of data storage and storage densities (multilevel memory states), are required to meet the criteria of practical data storage technology. Combining large-area, high-quality graphene from chemical vapour deposition process with photochromic molecules, meanwhile improving the reproducibility and cycle stability, are the obstacles to be tackled for application in transistors. An important concern in photochromic carbon-based nanodevices is whether the observed phenomena are truly due to the effects of photochromes or just a result of extrinsic switching. Therefore, control experiments should be always performed. A more delicate design via incorporating enhanced components combining photochromic molecules (for example, synthetic molecular motors^129^) with carbon-based nanomaterials is needed to realize controlled motion on a surface. Developing advanced imaging technique including scanning probe microscopies, fluorescence microscopy and nanoscopies is indispensable to observe the motion of nanovehicles at different length scales, from the sub-nanometer to the hundreds of micrometres scale. For practical applications in sensing, increasing the sensitivity, response speed and selectivity is a prerequisite. Multi-analyte sensing is a largely unexplored application in which photochromic carbon-based nanomaterials can hold great potential. Other photochromic molecules such as phenoxyquinone^130^ (Fig. 6) or dithienylethene derivatives could be also applied together with carbon-based nanomaterials for sensing.

How soon do we expect to see these developments? Basic research on constructing photochromic carbon-based nanomaterials with *ad hoc* properties is just starting, and further detailed studies are needed. The number of photochromic carbon-based nanomaterials is still limited, compared with the large amount of photochromic molecular systems which have been synthesized and investigated. However, one should bear in mind that some photochromic molecules seem not ideal to be coupled with carbon-based nanomaterials: for example, photochromic molecules relying on cycloadditions may react with the double bonds of carbon-based nanomaterials therefore losing the switching function. Besides the most widely used photochromic molecules discussed above, other photochromic systems such as dihydroazulene, fulgides/fulgimides, chromenes, hemithioindigo, retinal, quinones and so on, are also promising candidates to be coupled with carbon-based nanomaterials for specific applications. For instance, retinal or hemithioindigo combined with carbon-based nanomaterials could be useful for biological applications. Currently, the possibility of switching the magnetic properties of functional (nano)materials is gathering an increased attention. Photo-generated radical compounds^131^ or photo-induced valence tautomeric metal complexes^132^ are normally accompanied by reversible and distinct changes in their physical properties especially in magnetic property (Fig. 6). Combing them with carbon-based nanomaterials holds the potential to reversibly tune the magnetic properties of both components, and can be key for applications in spintronics. A greater attention should be also paid to design hybrid systems that can be switched between more than two states using different stimuli. For the development of useful and practical photochromic carbon-based nanomaterials, we must further understand the mechanisms that induce these phenomena to establish a true design strategy of the systems. All these studies will promote the design of well-defined structures and their self-assembly behaviour for novel photochromic carbon-based nanomaterials, ultimately paving the way towards new generation of responsive multifunctional (nano)materials.

## Additional information

**How to cite this article:** Zhang, X. *et al*. Coupling carbon nanomaterials with photochromic molecules for the generation of optically responsive materials. *Nat. Commun.* 7:11118 doi: 10.1038/ncomms11118 (2016).

## Figures and Tables

**Figure 1:**
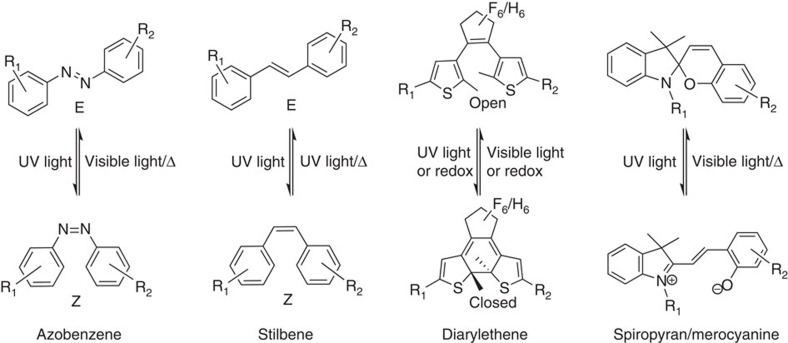
Chemical structures of the most widely used photochromic molecules. Azobenzenes, stilbenes and spiropyrans can be reverted either photochemically or thermally, while diarylethenes can be switched between open and closed form photochemically or electrochemically.

**Table 1:** Basic properties of the most widely used photochromic molecules.

Name	Isomerization process	Isomers	Properties of isomerization
Azobenzene	π–π* (*E* to *Z*)n-π* (*Z* to *E*)	*E* (nearly planar)*Z* (bent conformation)*E* is more stable than *Z*	Large conformational change; Medium change in dipole moment; Low rate of photobleaching
Stilbene	π–π* (*E* to *Z*, π* is metastable)π-π* (*Z* to *E*)	*E*/Z*E* is more stable than *Z*	Tendency of irreversible cyclization/oxidation in the *Z* form
Diarylethene	Photochemically or electrochemically induced cyclization	Open/closedBistable	Changes in conductance, fluorescence and so on;Fast photo-isomerization;Very-high fatigue resistance;
Spiropyran	Electrocyclic cleavage of the C-spiro-O bond	Spiropyran/merocyanineSpiropyran is more stable than merocyanineFast thermal back reaction	Colourless to colourful;Large change in dipole moment

**Table 2:** Modulated properties of photochromic carbon-based nanomaterials.

Function	Composition	Modulation range	References
Dipole moment	Azobenzene/CNTs	From 9 to 6 Debye	90
	Spiropyran/CNTs	A change up to 24.1 Debye	91
	Azobenzene/mechanical exfoliated graphene	From 9 to 6 Debye	92
	Azobenzene—chemically rGO	A change up to 3 Debye	97
	Spiropyran/chemically rGO	From 4 to 20 Debye	95
Current change	Dithienylethene-CNTs	*I*_on_/*I*_off_ ratio >10^5^	82
	Au/Azobenzene/mechanical exfoliated graphene	*I*_on_/*I*_off_ ratio of ∼100	87
	Azobenzene-graphene	*I*_on_/*I*_off_ ratio of >100 (pH change)	84
	Au/dihydroazulene/thermally rGO	Average *I*_on_/*I*_off_ ratio of ∼5–7	85
Charge transfer/separation	Dithienylethene-porphyrin-fullerene	Photoinduced electron transfer (25 ps) to singlet-singlet energy transfer (2.3 ps)	73
	Azobenzene-fullerene-porphyrin	Photo-induced intramolecular charge separation much faster than photo-isomerization	75
	Dihydroindolizine-porphyrin-fullerene	Quantum yield of electron transfer from 82 to 27%	76
	Dithienylethene-fullerene- tetrathiafulvalene	Reduced charge-separated state lifetime (from open to closed form)	78
Charge transport	Spiropyran/chemically rGO	Hole mobility from 2.9 to 2.2 cm^2^ V^−1^ s^−1^, electron mobility remains almost constant at 2.6 cm^2^ V^−1^ s^−1^	95
	Spiropyran/graphene grown by chemical vapour deposition	Hole and electron mobility from 508.8 to 392.4, and from 428.4 to 301.2 cm^2^ V^−1 ^s^−1^, respectively	94
	Azobenzene/mechanical exfoliated graphene	Charge carrier concentration from ∼5 × 10^13^ to ∼4 × 10^13^ cm^−2^ (2.8 × 10^12 ^cm^−2^ for the other method)	93
	Azobenzene/mechanical exfoliated graphene	Hole Concentrations from 4.3 × 10^12^ to 3.5 × 10^12^ cm^−2^	92
Magnetism	Azobenzene/graphene	0.8 μ_B_ (*E*)0.0 μ_B_ (*Z*)	71
Photoconductivity	Hexabenzocoronene-Dithienylethene	4.9 × 10^−3^ cm^2^V^−1^ s^−1^ (closed form)9.6 × 10^−4^ cm^2^V^−1^ s^−1^ (open form)	72
Fluorescence	Carbon nanodots-spiropyran	5 times change in intensity between closed and open form	55
	Carbon nanodots-spiropyran polymer	Reversible switching between blue-green and red	68
Dispersibility	Dendritic azobenzene/CNTs	Bundling–debundling	34
Morphology	Azobenzene-fullerene	Tunable 1D, 2D, 3D nanostructures	70
	Azo-polymer/mechanical exfoliated multilayer graphene	Internal pressure exceeding 1 GPa	118

CNT, carbon nanotube; rGO, reduced graphene oxide (the reduction can be done chemically, electrochemically or thermally and so on); 1D, one dimensional; 2D, two dimensional; 3D, three dimensional.

## References

[b1] Kinoshita K. Carbon: Electrochemical and Physicochemical Properties John Wiley Sons (1988).

[b2] Balandin A. A. Thermal properties of graphene and nanostructured carbon materials. Nat. Mater. 10, 569–581 (2011). 21778997 10.1038/nmat3064

[b3] Pierson H. O. Handbook of Carbon, Graphite, Diamond and Fullerenes Noyes Publications (1993).

[b4] Kroto H. W., Heath J. R., Obrien S. C., Curl R. F. & Smalley R. E. C_60_: buckminsterfullerene. Nature 318, 162–163 (1985).

[b5] Iijima S. Helical microtubules of graphitic carbon. Nature 354, 56–58 (1991).

[b6] Geim A. K. & Novoselov K. S. The rise of graphene. Nat. Mater. 6, 183–191 (2007). 17330084 10.1038/nmat1849

[b7] Novoselov K. S. . Electric field effect in atomically thin carbon films. Science 306, 666–669 (2004). 15499015 10.1126/science.1102896

[b8] Frackowiak E. & Béguinb F. Carbon materials for the electrochemical storage of energy in capacitors. Carbon 39, 937–950 (2001).

[b9] Tasis D., Tagmatarchis N., Bianco A. & Prato M. Chemistry of carbon nanotubes. Chem. Rev. 106, 1105–1136 (2006). 16522018 10.1021/cr050569o

[b10] Dubacheva G. V., Liang C. K. & Bassani D. M. Functional monolayers from carbon nanostructures–fullerenes, carbon nanotubes, and graphene–as novel materials for solar energy conversion. Coord. Chem. Rev. 256, 2628–2639 (2012).

[b11] Avouris P., Chen Z. & Perebeinos V. Carbon-based electronics. Nat. Nanotechnol. 2, 605–615 (2007). 18654384 10.1038/nnano.2007.300

[b12] Brabec C. J. . Polymer–fullerene bulk-heterojunction solar cells. Adv. Mater. 22, 3839–3856 (2010). 20717982 10.1002/adma.200903697

[b13] Baughman R. H., Zakhidov A. A. & de Heer W. A. Carbon nanotubes--the route toward applications. Science 297, 787–792 (2002). 12161643 10.1126/science.1060928

[b14] Wu D. Q., Zhang F., Liang H. W. & Feng X. L. Nanocomposites and macroscopic materials: Assembly of chemically modified graphene sheets. Chem. Soc. Rev. 41, 6160–6177 (2012). 22875044 10.1039/c2cs35179j

[b15] Wu J., Pisula W. & Müllen K. Graphenes as potential material for electronics. Chem. Rev. 107, 718–747 (2007). 17291049 10.1021/cr068010r

[b16] Stankovich S. . Graphene-based composite materials. Nature 442, 282–286 (2006). 16855586 10.1038/nature04969

[b17] Han W., Kawakami R. K., Gmitra M. & Fabian J. Graphene spintronics. Nat. Nanotechnol. 9, 324–340 (2014). 10.1038/nnano.2014.214 25286274

[b18] Li H., Kang Z., Liu Y. & Lee S.-T. Carbon nanodots: synthesis, properties and applications. J. Mater. Chem. 22, 24230 (2012).

[b19] Sazonova V. . A tunable carbon nanotube electromechanical oscillator. Nature 431, 284–287 (2004). 15372026 10.1038/nature02905

[b20] Kucharski T. J. . Templated assembly of photoswitches significantly increases the energy-storage capacity of solar thermal fuels. Nat. Chem. 6, 441–447 (2014) ***This paper gives an experimental study on photochromic molecules modified carbon nanotubes for solar thermal storage***. 24755597 10.1038/nchem.1918

[b21] del Valle M., Gutiérrez R., Tejedor C. & Cuniberti G. Tuning the conductance of a molecular switch. Nat. Nanotechnol. 2, 176–179 (2007) ***This paper is a theoretical study of carbon nanotube-photochromic molecule-carbon nanotube junctions***. 18654249 10.1038/nnano.2007.38

[b22] Bouas-laurent H. & Dürr H. Organic photochromism. Pure Appl. Chem. 73, 639–665 (2001).

[b23] Rau H., Rebek J. F. (eds). Photochemistry and Photophysics 2, 119CRC Press (1990).

[b24] Tian H. & Yang S. J. Recent progresses on diarylethene based photochromic switches. Chem. Soc. Rev. 33, 85–97 (2004). 14767504 10.1039/b302356g

[b25] Lukyanov B. S. & Lukyanova M. B. Spiropyrans: synthesis, properties, and application. Chem. Heterocycl. Compd. 41, 281–311 (2005).

[b26] Oudar J. L. Optical nonlinearities of conjugated molecules. Stilbene derivatives and highly polar aromatic compounds. J. Chem. Phys. 67, 446–457 (1977).

[b27] Klajn R. Spiropyran-based dynamic materials. Chem. Soc. Rev. 43, 148–184 (2014). 23979515 10.1039/c3cs60181a

[b28] Durr H., Bouas-Laurent H. (eds). Photochromism: Molecules and Systems Elsevier (2003).

[b29] El'tsov A. V. (ed.). Organic Photochromes Plenum Press (1990).

[b30] Brown G. H. (ed.). Photochromism in Techniques of Chemistry Wiley-Interscience (1971).

[b31] Feringa B. L., Browne W. R. (eds)). Molecular Switches Wiley−VCH: Weinheim (2011).

[b32] McGee D. J. . Molecular orientation and photoswitching kinetics on single-walled carbon nanotubes by optical second harmonic generation. Appl. Phys. Lett. 101, 264101 (2012).

[b33] Maggini L. . Azobenzene-based supramolecular polymers for processing MWCNTs. Nanoscale 5, 634–645 (2013). 23223852 10.1039/c2nr33358a

[b34] Kördel C. . Controlled reversible debundling of single-walled carbon nanotubes by photo-switchable dendritic surfactants. Nanoscale 5, 3029–3031 (2012). 10.1039/c2nr30305a 22504733

[b35] Bluemmel P., Setaro A., Popeney C. S., Haag R. & Reich S. Dispersion of carbon nanotubes using an azobenzene derivative. Phys. Status Solidi B 247, 2891–2894 (2010).

[b36] Vijayakumar C., Balan B., Kim M.-J. & Takeuchi M. Noncovalent functionalization of SWNTs with azobenzene-containing polymers: solubility, stability, and enhancement of photoresponsive properties. J. Phys. Chem. C 115, 4533–4539 (2011).

[b37] Huang C. . Spectroscopic properties of nanotube-chromophore hybrids. ACS Nano 5, 7767–7774 (2011). 21919456 10.1021/nn202725g

[b38] Bluemmel P., Setaro A., Maity C., Hecht S. & Reich S. Designing a spiropyran-based molecular switch for carbon nanotube functionalization: influence of anchor groups and tube–switch separation. Phys. Status Solidi B 249, 2479–2482 (2012).

[b39] Setaro A., Bluemmel P., Maity C., Hecht S. & Reich S. Non-covalent functionalization of individual nanotubes with spiropyran-based molecular switches. Adv. Funct. Mater. 22, 2425–2431 (2012).

[b40] Bluemmel P., Setaro A., Maity C., Hecht S. & Reich S. Tuning the interaction between carbon nanotubes and dipole switches-the influence of the change of the nanotube–spiropyran distance. J. Phys. Condens. Matter 24, 394005 (2012). 22964884 10.1088/0953-8984/24/39/394005

[b41] Imin P., Imit M. & Adronov A. Supramolecular functionalization of single-walled carbon nanotubes (SWNTs) with a photoisomerizable conjugated polymer. Macromolecules 45, 5045–5050 (2012).

[b42] Malic E. . Microscopic model of the optical absorption of carbon nanotubes functionalized with molecular spiropyran photoswitches. Phys. Rev. Lett. 106, 097401 (2011). 21405650 10.1103/PhysRevLett.106.097401

[b43] Setaro A., Kreft S. K., Petersen M. Å., Nielsen M. B. & Reich S. Optical properties of carbon nanotubes coated with orthogonal dipole switches. Phys. Status Solidi B 251, 2356–2359 (2014).

[b44] Kreft S. K., Petersen M. Å., Nielsen M. B., Reich S. & Setaro A. Isomerization of orthogonal molecular switches encapsulated within micelles solubilizing carbon nanotubes. J. Phys. Chem. C 119, 15731–15734 (2015).

[b45] Perry A., Green S. J., Horsell D. W., Hornett S. M. & Wood M. E. A pyrene-appended spiropyran for selective photo-switchable binding of Zn(II): UV-visible and fluorescence spectroscopy studies of binding and non-covalent attachment to graphene, graphene oxide and carbon nanotubes. Tetrahedron 71, 6776–6783 (2015).

[b46] Song Y. . Noncovalent functionalization of graphene with spiropyran molecular switches *via* a specially designed perylenediimide sdsorption anchor. Chem. Lett. 43, 868–870 (2014).

[b47] Chen S. . A cationic azobenzene-surfactant-modified graphene hybrid: unique photoresponse and electrochemical behavior. Nanoscale 7, 19673–19686 (2015). 26553111 10.1039/c5nr04646g

[b48] Feng Y. . Synthesis of photoresponsive azobenzene chromophore-modified multi-walled carbon nanotubes. Carbon 45, 2445–2448 (2007).

[b49] Feng Y. . Photoinduced anisotropic response of azobenzene chromophore functionalized multiwalled carbon nanotubes. J. Appl. Phys. 102, 053102 (2007).

[b50] Feng Y., Zhang X., Ding X. & Feng W. A light-driven reversible conductance switch based on a few-walled carbon nanotube-azobenzene hybrid linked by a flexible spacer. Carbon 48, 3091–3096 (2010).

[b51] Del Canto E., Flavin K., Natali M., Perova T. & Giordani S. Functionalization of single-walled carbon nanotubes with optically switchable spiropyrans. Carbon 48, 2815–2824 (2010).

[b52] Del Canto E., Natali M., Movia D. & Giordani S. Photo-controlled release of zinc metal ions by spiropyran receptors anchored to single-walled carbon nanotubes. Phys. Chem. Chem. Phys. 14, 6034–6043 (2012). 22446851 10.1039/c2cp40275k

[b53] Zhang X., Feng Y., Lv P., Shen Y. & Feng W. Enhanced reversible photoswitching of azobenzene-functionalized graphene oxide hybrids. Langmuir 26, 18508–18511 (2010). 21058679 10.1021/la1037537

[b54] Zhang X., Feng Y., Huang D., Li Y. & Feng W. Investigation of optical modulated conductance effects based on a graphene oxide–azobenzene hybrid. Carbon 48, 3236–3241 (2010).

[b55] Liao B. . Reversible fluorescence modulation of spiropyran-functionalized carbon nanoparticles. J. Mater. Chem. C 1, 3716–3721 (2013).

[b56] Wang Q. . Azobenzene dendronized carbon nanoparticles-the effect of light antenna. RSC Adv. 4, 18193–18197 (2014).

[b57] Khairutdinov R. F., Itkis M. E. & Haddon R. C. Light modulation of electronic transitions in semiconducting single wall carbon nanotubes. Nano Lett. 4, 1529–1533 (2004).

[b58] Cheong I. W., Wang S., Ki H. S. & Kim S.-H. Photoresponsive conductance switching of multi-walled carbon nanotubes bearing covalently linked spironaphthoxazine. Curr. Appl. Phys. 9, 1269–1271 (2009).

[b59] Devi R., Prabhavathi G., Yamuna R., Ramakrishnan S. & Kothurkar N. K. Synthesis, characterization and photoluminescence properties of graphene oxide functionalized with azo molecules. J. Chem. Sci. 126, 75–83 (2014).

[b60] Kay K.-Y., Han K.-J., Yu Y.-J. & Park Y. D. Dendritic fullerenes (C_60_) with photoresponsive azobenzene groups. Tetrahedron Lett. 43, 5053–5056 (2002).

[b61] Xu J.-H., Li Y.-L. & Zhu D. B. Synthesis of a new spiropyran based on [60]fullerene unit. Synth. Commun. 32, 1647–1652 (2002).

[b62] Schuster D. I. . Synthesis, photochemistry and photophysics of stilbene-derivatized fullerenes. Photochem. Photobiol. Sci. 2, 315–321 (2003). 12713233 10.1039/b211059h

[b63] Yan H. . A photoswitchable [2]rotaxane array on graphene oxide. Asian J. Org. Chem. 1, 314–318 (2012).

[b64] Wang Z., Li Z.-X. & Liu Z. Photostimulated reversible attachment of gold nanoparticles on multiwalled carbon nanotubes. J. Phys. Chem. C 113, 3899–3902 (2009).

[b65] Sadowska K. . Synthesis, characterization, and electrochemical testing of carbon nanotubes derivatized with azobenzene and anthraquinone. Carbon 47, 1501–1510 (2009).

[b66] Hu T., Xie H., Chen L., Zhong G. & Zhang H. Preparation and orientation behavior of multi-walled carbon nanotubes grafted with a side-chain azobenzene liquid crystalline polymer. Polym. Int. 60, 93–101 (2011).

[b67] Wang D., Ye G., Wang X. & Wang X. Graphene functionalized with azo polymer brushes: surface-initiated polymerization and photoresponsive properties. Adv. Mater. 23, 1122–1125 (2011). 21360765 10.1002/adma.201003653

[b68] Liao B. . The carbon nanoparticles grafted with copolymers of styrene and spiropyran with reversibly photoswitchable fluorescence. Carbon 91, 30–37 (2015).

[b69] Yang Y. . Structures and photoresponsive behaviors of multiwalled carbon nanotubes grafted by polyurethanes containing azobenzene side-chains. J. Phys. Chem. C 111, 11231–11239 (2007).

[b70] Kumar K. S. & Patnaik A. Tunable one-, two-, and three-dimensional self-assemblies from an acceptor-donor fullerene-N,N-dimethylaminoazobenzene dyad: interfacial geometry and temporal evolution. Langmuir 27, 11017–11025 (2011). 21766824 10.1021/la201849u

[b71] Nurbawono A. & Zhang C. Reversible magnetism switching in graphene-based systems *via* the decoration of photochromic molecules. Appl. Phys. Lett. 103, 203110 (2013).

[b72] He Y. . Hexabenzocoronene graphitic nanotube appended with dithienylethene pendants: hotochromism for the modulation of photoconductivity. Adv. Mater. 22, 829–832 (2010). 20217792 10.1002/adma.200902601

[b73] Liddell P. A., Kodis G., Moore A. L., Moore T. A. & Gust D. Photonic switching of photoinduced electron transfer in a dithienylethene–porphyrin–fullerene triad molecule. J. Am. Chem. Soc. 124, 7668–7669 (2002). 12083915 10.1021/ja026327c

[b74] Straight S. D. . Photochromic control of photoinduced electron transfer. Molecular double-throw switch. J. Am. Chem. Soc. 127, 2717–2724 (2005). 15725029 10.1021/ja044128i

[b75] Schuster D. I. . Azobenzene-linked porphyrin–fullerene dyads. J. Am. Chem. Soc. 129, 15973–15982 (2007). 18052375 10.1021/ja074684n

[b76] Straight S. D. . Self-regulation of photoinduced electron transfer by a molecular nonlinear transducer. Nat. Nanotechnol. 3, 280–283 (2008) ***The paper presented an artificial molecular system based on fullerene-porphyrin-photochromic molecule with self-regulation character resembling green plants***. 18654524 10.1038/nnano.2008.97

[b77] Kumar K. S. & Patnaik A. Solvent-polarity-tunable dimeric association of a fullerene (C_60_)-N,N-dimethylaminoazobenzene dyad: modulated electronic coupling of the azo chromophore with a substituted 3D fullerene. Chem. Eur. J. 17, 5327–5343 (2011). 21404350 10.1002/chem.201002981

[b78] Castellanos S. . Gating charge recombination rates through dynamic bridges in tetrathiafulvalene-fullerene architectures. Angew. Chem. Int. Ed. 52, 13985–13990 (2013). 10.1002/anie.201306183 24214915

[b79] Jia C. & Guo X. Molecule–electrode interfaces in molecular electronic devices. Chem. Soc. Rev. 42, 5642–5660 (2013). 23571285 10.1039/c3cs35527f

[b80] Ashraf M. K., Bruque N. A., Tan J. L., Beran G. J. O. & Lake R. K. Conductance switching in diarylethenes bridging carbon nanotubes. J. Chem. Phys. 134, 024524 (2011). 21241137 10.1063/1.3528118

[b81] Motta C., Trioni M. I., Brivio G. P. & Sebastian K. L. Conductance of a photochromic molecular switch with graphene leads. Phys. Rev. B 84, 113408 (2011).

[b82] Whalley A. C., Steigerwald M. L., Guo X. & Nuckolls C. Reversible switching in molecular electronic devices. J. Am. Chem. Soc. 129, 12590–12591 (2007) ***This paper is an experimental study on carbon nanotube-photochromic molecule-carbon nanotube junctions***. 17902658 10.1021/ja073127y

[b83] Jia C. . Conductance switching and mechanisms in single-molecule junctions. Angew. Chem. Int. Ed. 52, 8666–8670 (2013). 10.1002/anie.201304301 23818452

[b84] Cao Y., Dong S., Liu S., Liu Z. & Guo X. Toward functional molecular devices based on graphene–molecule junctions. Angew. Chem. Int. Ed. 52, 3906–3910 (2013). 10.1002/anie.201208210 23460546

[b85] Li T. . Ultrathin reduced graphene oxide films as transparent top-contacts for light switchable solid-state molecular junctions. Adv. Mater. 25, 4164–4170 (2013). 23765569 10.1002/adma.201300607

[b86] Seo S., Min M., Lee S. M. & Lee H. Photo-switchable molecular monolayer anchored between highly transparent and flexible graphene electrodes. Nat. Commun. 4, 1920 (2013) ***This paper reports a study combining both covalent and noncovalent approaches in a single graphene-photochromic molecules-graphene junction***. 23715279 10.1038/ncomms2937

[b87] Margapoti E. . Emergence of photoswitchable states in a graphene-azobenzene-Au platform. Nano Lett. 14, 6823–6827 (2014). 25414977 10.1021/nl503681z

[b88] Kim D. . Reversible switching phenomenon in diarylethene molecular devices with reduced graphene oxide electrodes on flexible substrates. Adv. Funct. Mater. 25, 5918–5923 (2015).

[b89] Guo X., Huang L., O'Brien S., Kim P. & Nuckolls C. Directing and sensing changes in molecular conformation on individual carbon nanotube field effect transistors. J. Am. Chem. Soc. 127, 15045–15047 (2005). 16248641 10.1021/ja054335y

[b90] Simmons J. M. . Optically modulated conduction in chromophore-functionalized single-wall carbon nanotubes. Phys. Rev. Lett. 98, 086802 (2007). 17359117 10.1103/PhysRevLett.98.086802

[b91] Zhao Y. . Functionalization of single-wall carbon nanotubes with chromophores of opposite internal dipole orientation. ACS Appl. Mater. Interfaces 5, 9355–9361 (2013). 24060382 10.1021/am4024753

[b92] Kim M., Safron N. S., Huang C., Arnold M. S. & Gopalan P. Light-driven reversible modulation of doping in graphene. Nano Lett. 12, 182–187 (2012). 22149166 10.1021/nl2032734

[b93] Peimyoo N. . Photocontrolled molecular structural transition and doping in graphene. ACS Nano 6, 8878–8886 (2012). 22966836 10.1021/nn302876w

[b94] Jang A. . Reversibly light-modulated dirac point of graphene functionalized with spiropyran. ACS Nano 6, 9207–9213 (2012). 22980316 10.1021/nn303539y

[b95] Joo P., Kim B. J., Jeon E. K., Cho J. H. & Kim B.-S. Optical switching of the Dirac point in graphene multilayer field-effect transistors functionalized with spiropyran. Chem. Commun. 48, 10978–10980 (2012). 10.1039/c2cc35933b 23032739

[b96] Sciascia C. . Light-controlled resistance modulation in a photochromic diarylethene–carbon nanotube blend. J. Phys. Chem. C 116, 19483–19489 (2012).

[b97] Nguyen P. . Covalent functionalization of dipole-modulating molecules on trilayer graphene: an avenue for graphene-interfaced molecular machines. Small 9, 3823–3828 (2013). 23713056 10.1002/smll.201300857

[b98] Börjesson K. . Optically switchable transistors comprising a hybrid photochromic molecule/n-type organic active layer. J. Mater. Chem. C 3, 4156–4161 (2015).

[b99] Malic E. . Carbon nanotubes as substrates for molecular spiropyran-based switches. J. Phys. Condens. Matter 24, 394006 (2012). 22964905 10.1088/0953-8984/24/39/394006

[b100] Zhou X. . Color detection using chromophore-nanotube hybrid devices. Nano Lett. 9, 1028–1033 (2009). 19206226 10.1021/nl8032922 PMC3258674

[b101] Mativetsky J. M. . Azobenzenes as light-controlled molecular electronic switches in nanoscale metal−molecule−metal junctions. J. Am. Chem. Soc. 130, 9192–9193 (2008). 18576645 10.1021/ja8018093

[b102] Crivillers N., Orgiu E., Reinders F., Mayor M. & Samorì P. Optical modulation of the charge injection in an organic field-effect transistor based on photochromic self-assembled-monolayer-functionalized electrodes. Adv. Mater. 23, 1447–1452 (2011). 21433111 10.1002/adma.201003736

[b103] Kolpak A. M. & Grossman J. C. Hybrid chromophore-template nanostructures: A customizable platform material for solar energy storage and conversion. J. Chem. Phys. 138, 034303 (2013). 23343272 10.1063/1.4773306

[b104] Kolpak A. M. & Grossman J. C. Azobenzene-functionalized carbon nanotubes as high-energy density solar thermal fuels. Nano Lett. 11, 3156–3162 (2011) ***This paper reports a theoretical study on photochromic carbon-based nanomaterails for solar thermal storage***. 21688811 10.1021/nl201357n

[b105] Feng Y. . Covalent functionalization of graphene by azobenzene with molecular hydrogen bonds for long-term solar thermal storage. Sci. Rep. 3, 3260 (2013). 24247355 10.1038/srep03260 PMC3832871

[b106] Luo W. . A high energy density azobenzene/graphene hybrid: a nano-templated platform for solar thermal storage. J. Mater. Chem. A 3, 11787–11795 (2015).

[b107] Luo W. . High-energy, stable and recycled molecular solar thermal storage materials using AZO/graphene hybrids by optimizing hydrogen bonds. Nanoscale 7, 16214–16221 (2015). 26289389 10.1039/c5nr03558a

[b108] Min M., Seo S., Lee S. M. & Lee H. Voltage-controlled nonvolatile molecular memory of an azobenzene monolayer through solution-processed reduced graphene oxide contacts. Adv. Mater. 25, 7045–7050 (2013). 24133048 10.1002/adma.201303335

[b109] Lin C.-Y., Chang C.-S., Lin J. H., Hsu C.-C. & Chien F. S.-S. Optical controlled graphene-based nonvolatile ternary-logic transistor with azobenzene copolymer. Appl. Phys. Lett. 102, 013505 (2013).

[b110] Song Y., Xu C., Wei W., Ren J. & Qu X. Light regulation of peroxidase activity by spiropyran functionalized carbon nanotubes used for label-free colorimetric detection of lysozyme. Chem. Commun. 47, 9083–9085 (2011). 10.1039/c1cc13279b 21748199

[b111] Lee J. H., Jaworski J. & Jung J. H. Luminescent metal–organic framework-functionalized graphene oxide nanocomposites and the reversible detection of high explosives. Nanoscale 5, 8533–8540 (2013). 23892560 10.1039/c3nr01439h

[b112] Li Y. . Self-assembly of graphene oxide with a silyl-appended spiropyran dye for rapid and sensitive colorimetric detection of fluoride ions. Anal. Chem. 85, 11456–11463 (2013). 24164279 10.1021/ac402592c

[b113] Wang C. . Graphene oxide assisted fluorescent chemodosimeter for high-performance sensing and bioimaging of fluoride ions. ACS Appl. Mater. Interfaces 6, 9768–9775 (2015). 24837223 10.1021/am502142d

[b114] Nahain A.-A., Lee J.-E., Jeong J. H. & Park S. Y. Photoresponsive fluorescent reduced graphene oxide by spiropyran conjugated hyaluronic acid for *in vivo* imaging and target delivery. Biomacromolecules 14, 4082–4090 (2013). 24106989 10.1021/bm4012166

[b115] Sharker S. M. . Photo- and pH-tunable multicolor fluorescent nanoparticle-based spiropyran- and BODIPY-conjugated polymer with graphene oxide. Chem. Asian J. 9, 2921–2927 (2014). 25056486 10.1002/asia.201402399

[b116] Basuki S. W., Schneider V., Strunskus T., Elbahri M. & Faupel F. Light-controlled conductance switching in azobenzene-containing MWCNT-polymer nanocomposites. ACS Appl. Mater. Interfaces 7, 11257–11262 (2015). 25961784 10.1021/acsami.5b01319

[b117] Schneidera V., Strunskusa T., Elbahrib M. & Faupel F. Light-induced conductance switching in azobenzene based near-percolated single wall carbon nanotube/polymer composites. Carbon 90, 94–101 (2015).

[b118] Florio G. D., Bründermann E., Yadavalli N. S., Santer S. & Havenith M. Graphene multilayer as nanosized optical strain gauge for polymer surface relief gratings. Nano Lett. 14, 5754–5760 (2014). 25244634 10.1021/nl502631s

[b119] Sasaki T. & Tour J. M. Synthesis of a new photoactive nanovehicle: a nanoworm. Org. Lett. 10, 897–900 (2008) ***This paper designs a photochromic carbon-based nanomaterial for nanovehicles***. 18260667 10.1021/ol703027h

[b120] Li M. . Site-selective fabrication of two-dimensional fullerene arrays by using a supramolecular template at the liquid-solid interface. Angew. Chem. Int. Ed. 47, 6717–6721 (2008). 10.1002/anie.200802518 18655080

[b121] Ding K., Grebel-Koehler D., Berger R., Müllen K. & Butt H.-J. Structure of self-assembled n-dodecyl substituted azobenzene poly(phenylene) dendrimers on graphite. J. Mater. Chem. 15, 3431–3436 (2005).

[b122] Ai M. . Optical switching studies of an azobenzene rigidly linked to a hexa-peri-hexabenzocoronene derivative in solution and at a solid-liquid interface. Appl. Phys. A 93, 277–283 (2008).

[b123] Wang D. & Wang X. Self-assembled graphene/azo polyelectrolyte multilayer film and its application in electrochemical energy storage device. Langmuir 27, 2007–2013 (2011). 21244083 10.1021/la1044128

[b124] Nguyen T.-T.-T. . A fluorescent, shape-persistent dendritic host with photoswitchable guest encapsulation and intramolecular energy transfer. J. Am. Chem. Soc. 133, 11194–11204 (2011). 21682280 10.1021/ja2022398

[b125] Nguyen T.-T.-T., Türp D., Wagner M. & Müllen K. Photoswitchable conductivity in a rigidly dendronized salt. Angew. Chem. Int. Ed. 52, 669–673 (2013). 10.1002/anie.201206010 23192942

[b126] Keirstead A. E. . Photochemical ‘triode' molecular signal transducer. J. Am. Chem. Soc. 132, 6588–6595 (2010). 20408535 10.1021/ja1019595

[b127] Neale N. R. Solar energy: packing heat. Nat. Chem. 6, 385–386 (2014). 24755588 10.1038/nchem.1933

[b128] Lennartson A., Roffey A. & Moth-Poulsen K. Designing photoswitches for molecular solar thermal energy storage. Tetrahedron Lett. 56, 1457–1465 (2015).

[b129] Koumura N., Zijlstra R. W. J., van Delden R. A., Harada N. & Feringa B. L. Light-driven monodirectional molecular rotor. Nature 401, 152–155 (1999). 10490022 10.1038/43646

[b130] Park I. S., Heo E.-J. & Kim J.-M. A photochromic phenoxyquinone based cyanide ion sensor. Tetrahedron Lett. 52, 2454–2457 (2011).

[b131] Xu L. . Photoinduced ground-state singlet biradical-novel insight into the photochromic compounds of biindenylidenediones. Chem. Commun. 2328–2329 (2002). 10.1039/b206876a 12430425

[b132] Sato O. . Photo-induced long-lived intramolecular electron transfer in a Co valence tautomeric complex. Chem. Lett. 30, 874–875 (2001).

